# Seasonal Dynamics of the Airborne Bacterial Community and Selected Viruses in a Children’s Daycare Center

**DOI:** 10.1371/journal.pone.0151004

**Published:** 2016-03-04

**Authors:** Aaron J. Prussin, Amit Vikram, Kyle J. Bibby, Linsey C. Marr

**Affiliations:** 1 Department of Civil and Environmental Engineering, Virginia Tech, Blacksburg, Virginia, United States of America; 2 Department of Civil and Environmental Engineering, University of Pittsburgh, Pittsburgh, Pennsylvania, United States of America; Peking University, CHINA

## Abstract

Children’s daycare centers appear to be hubs of respiratory infectious disease transmission, yet there is only limited information about the airborne microbial communities that are present in daycare centers. We have investigated the microbial community of the air in a daycare center, including seasonal dynamics in the bacterial community and the presence of specific viral pathogens. We collected filters from the heating, ventilation, and air conditioning (HVAC) system of a daycare center every two weeks over the course of a year. Amplifying and sequencing the 16S rRNA gene revealed that the air was dominated by Proteobacteria, Firmicutes, Actinobacteria, and Bacteroidetes that are commonly associated with the human skin flora. Clear seasonal differences in the microbial community were not evident; however, the community structure differed when the daycare center was closed and unoccupied for a 13-day period. These results suggest that human occupancy, rather than the environment, is the major driver in shaping the microbial community structure in the air of the daycare center. Using PCR for targeted viruses, we detected a seasonal pattern in the presence of respiratory syncytial virus that included the period of typical occurrence of the disease related to the virus; however, we did not detect the presence of adenovirus or rotavirus at any time.

## 1. Introduction

Understanding the microbial community structure of the built environment is important because humans spend >90% of their time in indoor environments [1], and evidence is accumulating that both the human and environmental microbiomes shape human health [2–4]. Studies have characterized the microbiome of indoor air in residences, health care facilities, university classrooms, offices, restrooms, subways, and other settings [5–11], but less is known about children’s daycare centers.

Over 8 million children attend daycare in organized facilities in the United States [12]. Children who attend daycare centers have a higher incidence of certain diseases and get sicker compared to children who do not attend daycare centers [13–18]. Thacker et al. [17] estimated that preschool-aged children who spend time in a daycare center are at 2–4 times greater risk for developing an infectious disease than are children cared for at home. Hurwitz et al. [19] reported an increased risk of respiratory illness in children attending daycare centers. Marbury et al. [20] found that daycare attendance is an important risk factor for lower respiratory illness and recurrent wheezing in young children. Effects extend to parents or guardians, who may lose an average of 13 workdays annually caring for children who are ill [21].

Models of the spread of diseases such as influenza in a community have shown that schools and childcare centers are hubs for transmission [22–27]. Many common respiratory illnesses have an airborne transmission route [28–31], so the air in a daycare center represents a unique opportunity for sampling certain pathogenic microbes. Respiratory syncytial virus (RSV), influenza, adenovirus, rhinovirus, rotavirus, and coxsackievirus A16 are commonly associated with disease outbreaks in daycare centers and can exist as bioaerosols [32–35]. For example, Yang et al. [36] found average concentrations of influenza A virus of 2 × 10^4^ genome copies per m^3^ of air in a daycare center in three samples that were positive for the virus; one sample was negative. Of course, the airborne microbiome encompasses all microorganisms, not just pathogenic ones. Prussin II et al. [37] reported total bacterial-like and viral-like bioaerosol concentrations in a daycare center to be 5.0 × 10^5^ and 4.5 × 10^5^, respectively.

Prior studies have investigated the microbiome of daycare centers. A study of five daycare centers and five elementary schools in South Korea found that the airborne bacterial community reflected both humans and the outdoor environment [38], and there was no correlation between specific taxa and temperature and relative humidity. In a study of four daycare classrooms in the US, researchers swabbed toys and furniture and found sequences that were related to *Pseudomonas* spp. and bacteria associated with humans, including some pathogens and opportunistic pathogens, and with wastewater sludge [39]. In these studies, sampling was limited to approximately two months [38] or did not include airborne microorganisms [39].

One question that has not yet been investigated, to our knowledge, is whether the airborne microbial community of a daycare center exhibits seasonal dynamics. The incidence of some diseases that occur commonly in children who attend daycare centers, such as hand, foot, and mouth disease (HFMD), influenza, and gastroenteritis caused by rotavirus, follows a seasonal pattern [35, 40–42]. Understanding how the airborne microbial community changes with season could provide insight into the epidemiology of seasonal infectious diseases [41]. Additionally, given the important role of viruses in respiratory disease outbreaks in daycare centers, studies examining viral bioaerosols have been especially limited, due to challenges in both sampling and data analysis [43, 44].

The objectives of this study are to determine the seasonal dynamics in the bacterial community structure and in the presence of selected pathogenic viruses in the air of a daycare center. We hypothesize that daycare centers harbor a unique and dynamic microbial community structure that is strongly influenced by occupancy. Additionally, we hypothesize that there are seasonal patterns in the community structure of airborne bacteria and the presence of selected pathogenic viruses. Our results provide novel information about the seasonal dynamics of airborne bacteria and viruses in a daycare center over a year-long period.

## 2. Materials and Methods

### 2.1 Bioaerosol Collection

We collected air samples continuously between January 2014 and February 2015 at a daycare center located in Blacksburg, Virginia, USA, with the center’s permission. Approximately 200 children of both genders from ages newborn to ~10 years old attend the center; the older children attend only in the early morning before school and the late afternoon after school. The center is open from 7:15 am to 5:45 pm Monday through Friday, and a typical day includes organized reading and artwork activities, indoor and outdoor play, snack time, lunch time, and nap time. The center’s windows remain closed, and the rooms are cleaned daily, including removal of garbage, vacuuming and mopping of floors, and cleaning of kitchen and bathroom surfaces. The center has a total floor area of 1187 m^2^ (12,800 ft^2^) split between two buildings, each of which is served by a 4-ton split-system heat pump rated at 2000 ft^3^ min^-1^ (Carrier). We installed a 50.8 cm × 50.8 cm pleated filter (Nordic Pure, Tulsa, OK) in a heating, ventilation, and air conditioning (HVAC) return duct that was located in the wall of a large, interior hallway connecting the entrance lobby to the kitchen and four children’s rooms, and we changed the filter once every two weeks. This return duct was the only one serving one entire building, and children, parents, and teachers walked past it regularly. The velocity in the duct, measured 5 cm above the centerline and 15 cm to the side of it, was 2 m s^-1^, producing a flow rate of ~0.6 m^3^ s^-1^ after accounting for the velocity profile in a square duct [45]. Between 23 December 2014 and 5 January 2015, the daycare center was closed for the winter holidays, and we collected a filter sample corresponding to this 13-day period when the building was unoccupied, except for occasional cleaning workers. Table 1 lists the dates of each sample.

**Table 1:** Sampling dates and average building parameters over the sampling period.

Sample Number	Start Date	End Date	Average Temperature (°C)	Average Relative Humidity (%)	% Time HVAC On
1	20 January 2014	03 February 2014	19.7	26.6	^D^
2	03 February 2014	17 February 2014	19.9	31.3	^D^
3	17 February 2014	03 March 2014	20.5	32.9	^D^
4	03 March 2014	17 March 2014	20.2	33.4	^D^
5	17 March 2014	31 March 2014	20.2	35.2	^D^
6	31 March 2014	14 April 2014	20.6	42.3	^D^
7	14 April 2014	28 April 2014	20.4	44.2	^D^
8	28 April 2014	12 May 2014	21.2	49.0	^D^
9	12 May 2014	27 May 2014^A^	21.0	49.6	^D^
10	27 May 2014^A^	09 June 2014	20.7	55.7	38%
11	09 June 2014	23 June 2014	20.9	54.0	47%
12	23 June 2014	07 July 2014	20.8	56.9	40%
13	07 July 2014	21 July 2014	20.7	57.1	40%
14	21 July 2014	04 August 2014	20.7	58.4	37%
15	04 August 2014	18 August 2014	20.5	62.7	29%
16	18 August 2014	02 September 2014^A^	20.7	59.8	38%
17	02 September 2014^A^	15 September 2014	20.5	64.2	33%
18	15 September 2014	29 September 2014	19.7	65.7	17%
19	29 September 2014	13 October 2014	19.0	65.1	13%
20	13 October 2014	27 October 2014	18.2	65.3	10%
21	27 October 2014	10 November 2014	18.0	55.1	21%
22	10 November 2014	24 November 2014	17.8	44.5	40%
23	24 November 2014	08 December 2014	17.0	53.0	29%
24	08 December 2014	23 December 2014^B^	17.6	45.5	46%
25^C^	23 December 2014^B^	05 January 2015	17.7	43.3	38%
26	05 January 2015	19 January 2015	17.9	37.9	60%
27	19 January 2015	02 February 2015	17.6	42.0	47%

^A^ HVAC filters changed on Tuesday because the daycare center was closed on Monday for a holiday.

^B^ HVAC filter changed on Tuesday to allow for a sample when the daycare center would be unoccupied.

^C^ Daycare center closed for the winter holidays, unoccupied except for occasional cleaning workers.

^D^ No data, as equipment to measure HVAC operation was not set up until 27 May 2014.

The HVAC filter had a Minimum Efficiency Reporting Value (MERV) rating of 14, meaning that its average particle collection efficiency was >98%, and its efficiency over the particle size range of 0.3 μm to 1.0 μm was 75–85% according to standards of the American Society of Heating, Refrigerating and Air-Conditioning Engineers. Efficiency for particles smaller than 0.3 μm would be higher because of particle removal by Brownian motion, although electrostatic charging of the filter media could also affect collection efficiency. After removing an exposed filter, we transported it immediately to the laboratory, cut it into ~8 cm × 8 cm squares under sterile conditions, placed them in a sterile breast milk bag (Lansinoh, Alexandria, VA), and froze them at -80°C until further processing.

We measured building parameters, including temperature, relative humidity, and HVAC operational status (i.e., on/off), in the duct where the filter was located using a HOBO weather station and data logger (Onset Computer Corporation, Bourne, MA). Table 1 shows the indoor conditions during each sampling period.

### 2.2 Bacteria Processing and Sequencing

To prepare samples for analysis of bacteria, we cut two or three squares (~8 cm × 8 cm) from each filter into smaller pieces (~2 cm^2^) and placed them into a 50-mL conical tube. To remove bacteria from the filter, we vigorously vortexed the filter pieces in ~30 mL of 0.02% Tween-80 in molecular biology grade water and then agitated them for ~15 min at 200 rpm [46]. It is possible that some bacteria were not dislodged from the filter, so results represent those that were successfully removed. We extracted bacterial DNA using the QIAamp DNA Mini Kit following the manufacturer’s protocol (Qiagen, Valencia, CA). An unexposed HVAC filter and a QIAamp DNA Mini Kit blank served as negative controls to indicate any microbial contamination in the filters and/or extraction kit. As previously described [47], we amplified DNA samples using barcoded 16S rRNA primers, purified using AMPure beads (Beckman Coulter, Pasadena, CA), and sequenced using a 300-cycle MiSeq V2 Nano kit (Illumina, San Diego, CA). Sequences are available on the MG-RAST server under accession number 4672709.3. There was no 16S rRNA amplification in either of the negative controls.

### 2.3 Bacteria Community Analysis

We performed sequence analysis using tools in the Quantitative Insights Into Microbial Ecology (QIIME) package [48]. We demultiplexed sequences and trimmed identifier primer sequences. We excluded sequences if they did not have an exact barcode match, were shorter than 200 base pairs (bp) in length, had ambiguous bases, or had a quality score below 20. We clustered sequences into operational taxonomic units (OTUs) at a minimum identity of 97% aligned against the Greengenes core set [49]. To determine alpha diversity within QIIME, we used single rarefaction. We conducted a two-dimensional principal coordinate analysis in QIIME to compare how closely communities from each sample were related. To assess differences and relationships between bacterial beta diversity and season or operational parameters, we implemented ANOSIM and ADONIS in QIIME 1.9.1 [48]. We compared pairwise UniFrac distances against season, temperature, relative humidity, and air velocity in the HVAC duct. We used 999 permutations to calculate p-values.

### 2.4 Virus Processing

To prepare samples for analysis of viruses, we cut two or three squares (~8 cm × 8 cm) from each filter into smaller pieces (~2 cm^2^) and placed them into a 50-mL conical tube. To remove viruses from the filter, we vigorously vortexed the filter pieces in ~20 mL of 3% beef extract and 0.05 M glycine in molecular biology grade water and then shook them for ~15 min at 200 rpm [46]. It is possible that some viruses were not dislodged from the filter, so results represent those that were successfully removed. We extracted viral nucleic acid using the QIAamp Viral RNA Mini Kit following the manufacturer’s protocol (Qiagen, Valencia, CA). An unexposed HVAC filter and a QIAamp DNA Mini Kit blank served as negative controls to indicate any microbial contamination in the filters and/or extraction kit. For nucleic acid stability, immediately following RNA extraction we converted samples to cDNA using the iScript cDNA Synthesis Kit (Bio-Rad, Hercules, CA). As rotavirus is a dsRNA virus, we had to separate the strands immediately prior to cDNA synthesis by combining extracted RNA and 100% DMSO at a 1:1 ratio and incubating at 95°C for 10 min. We froze samples at -20°C until polymerase chain reaction (PCR) analysis.

### 2.5 Virus PCR

We used PCR to determine the presence of the following viruses during each two-week sampling period: adenovirus, enterovirus-71, influenza A virus (IAV), respiratory syncytial virus (RSV), rhinovirus, and rotavirus. Table 2 shows forward and reverse primer sequences, including expected PCR amplicon length. We performed PCR in 25-μL volumes containing 5 μL of template cDNA or DNA, 12.5 μL of 2X Go Taq Green MasterMix (Promega, Madison, WI), between 200 nM and 500 nM forward and reverse primers depending on virus (Table 2), and nuclease-free water to bring the total volume up to 25 μL. Table in S1 Table presents PCR cycling conditions. We diluted cDNA and DNA templates 1:100 to minimize the effect of PCR inhibitors typically present in environmental samples.

**Table 2:** Primer sequences and expected amplicon lengths to detect the presence of specific viruses.

Virus	Forward Primer Sequence (PCR Reaction Concentration)	Reverse Primer Sequence (PCR Reaction Concentration)	Expected Amplicon Length (bp)	Ref.
Adenovirus	5'-CTGATGTACTACAACAGCACTGGCAACATGGG-3' (500 nM)	5'-GCGTTGCGGTGGTGGTTAAATGGGTTTACGTTGTCCAT-3' (500 nM)	605	[77]
Enterovirus-71	5'-ATAATAGCAYTRGCGGCAGCCCA-3' (500 nM)	5'-AGCTGTGCTATGTGAATTAGGAA-3' (500 nM)	317	[78]
IAV	5'-AAGACCAATCCTGTCACCTCTGA-3' (200 nM)	5'-CATTCTGTTGTATATGAGGCCCAT-3' (200 nM)	262	[36]
RSV	5'-TTGGATCTGCAATCGCCA-3' (300 nM)	5'-CTTTTGATCTTGTTCACTTCTCCTTCT-3' (300 nM)	75	[79]
Rhinovirus	5'-TGGACAGGGTGTGAAGAGC-3' (200 nM)	5'-CAAAGTAGTCGGTCCCATCC-3' (200 nM)	235	[80]
Rotavirus	5'-GCTATTAAAGGCTCAATGGCGTAC-3' (250 nM)	5'-GGATGTAGAATTGATGGATAATTG-3' (250 nM)	834	[81]

We ran PCR products on a 1.2% agarose gel for ~75 min at 90 V and stained the amplicons using SYBR Green I Nucleic Acid Gel Stain (ThermoFisher Scientific, Grand Island, NY). Through visualization of the gels using a Gel Doc XR+ System (Bio-rad, Hercules, CA), we analyzed samples for the presence or absence of a virus. We defined samples that produced a PCR product as containing the virus of interest. We included three negative controls (an unexposed filter, QIAamp Viral RNA Mini Kit blank, and PCR water blank) and purified RNA (converted to cDNA) or DNA extracted from each virus as positive controls.

## 3. Results and Discussion

### 3.1 Bacterial Community

After trimming, sorting, and quality control, there were 138,047 sequences with a median sequence length of 251 bases for analysis. The unexposed filter did not produce a detectable 16S rRNA amplicon, and thus we assumed background bacterial contamination on the HVAC filters was negligible. We classified samples collected between 17 March-9 June 2014 as ‘Spring’, 9 June- 2 September 2014 as ‘Summer’, 2 September-24 November 2014 as ‘Fall’, and 20 January-17 March 2014 and 24 November 2014–2 February 2015 as ‘Winter’. The sample collected between 23 December 2014–5 January 2015 corresponded to when the daycare center was closed, and thus we classified it as ‘Unoccupied’. We excluded the three samples collected between 20 January-3 February 2014, 3–17 February 2014, and 12–27 May 2014 from further analysis because the percentage of sequences that could not be assigned any taxonomy was 91%, 66%, and 67%, respectively. These percentages were much higher than the average unassigned rate of <3% across all other samples, indicating potential problems in the preparation and/or sequencing of these samples. An inherent limitation of the sequencing-based approach is that it does not discriminate between viable and non-viable organisms. Thus, the “community” that we describe is that defined by its genetic material.

**Fig 1:**
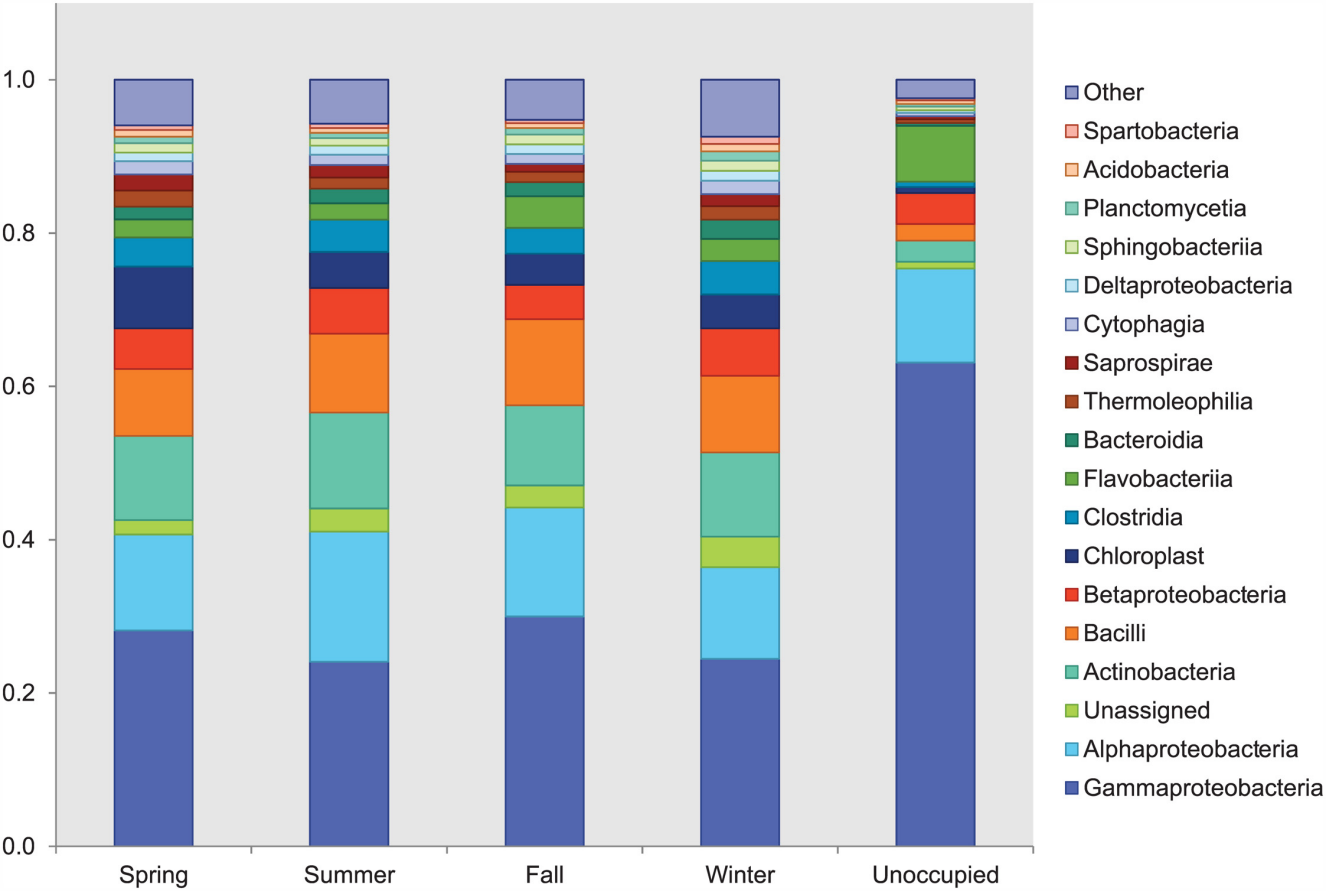
Phylogenetic classifications to the class level by season and when the center was unoccupied.

Taxonomic analysis revealed that bacterial bioaerosols were diverse yet were dominated (greater than 60% of sequences in samples) by the classes Gammaproteobacteria, Alphaproteobacteria, Actinobacteria, Bacilli, and Betaproteobacteria (Fig 1). Additionally, the dominant bacterial phyla were Proteobacteria, followed by Firmicutes, Actinobacteria, and Bacteroidetes, with year-long relative abundance averages of 49%, 14%, 13%, and 9%, respectively. Three of these—Actinobacteria, Proteobacteria, and Firmicutes—also were the dominant phyla in a metagenomic study of airborne microorganisms in daycare centers and schools in South Korea [38]. A study of the bacterial composition of dust in daycare centers in Finland showed that Gram-positive Bacilli and Actinomycetales (class Actinobacteria) dominated the airborne bacterial flora [50]. Prior studies of the microbiome in homes, office buildings, and classrooms have identified the same dominant phyla and classes [9, 51–53]. Given the current state of knowledge, the airborne microbiome of daycare centers appears to be similar to that of other indoor environments. It is possible that future tools may enable identification of unique features of the airborne microbiome of daycare centers, if they exist, but very carefully controlled studies would be required. A meta-analysis of 16 studies of the microbiome of the indoor environment found that technical variations between studies, such as geographic location and sample type, strongly affect comparisons of the microbial community [54].

The dominant phyla match the four that account for most of the typical human skin flora: Actinobacteria, Bacteroidetes, Firmicutes, and Proteobacteria [55]. The building where we collected our samples was occupied by ~100 children and their adult teachers at any given time. Humans shed 5 × 10^8^ cells day^-1^ [56]; thus human occupancy should have a dramatic impact on shaping the microbiome of the indoor environment. Qian et al. [10] measured bacterial bioaerosol emission rates of 3.7 × 10^7^ bacterial genome copies per person-hour in a classroom. As in other studies of densely occupied environments [9, 57], our results suggest that the occupants of the daycare, and not the outdoor environment or other sources, are the main driver of the community structure of airborne bacteria.

**Fig 2:**
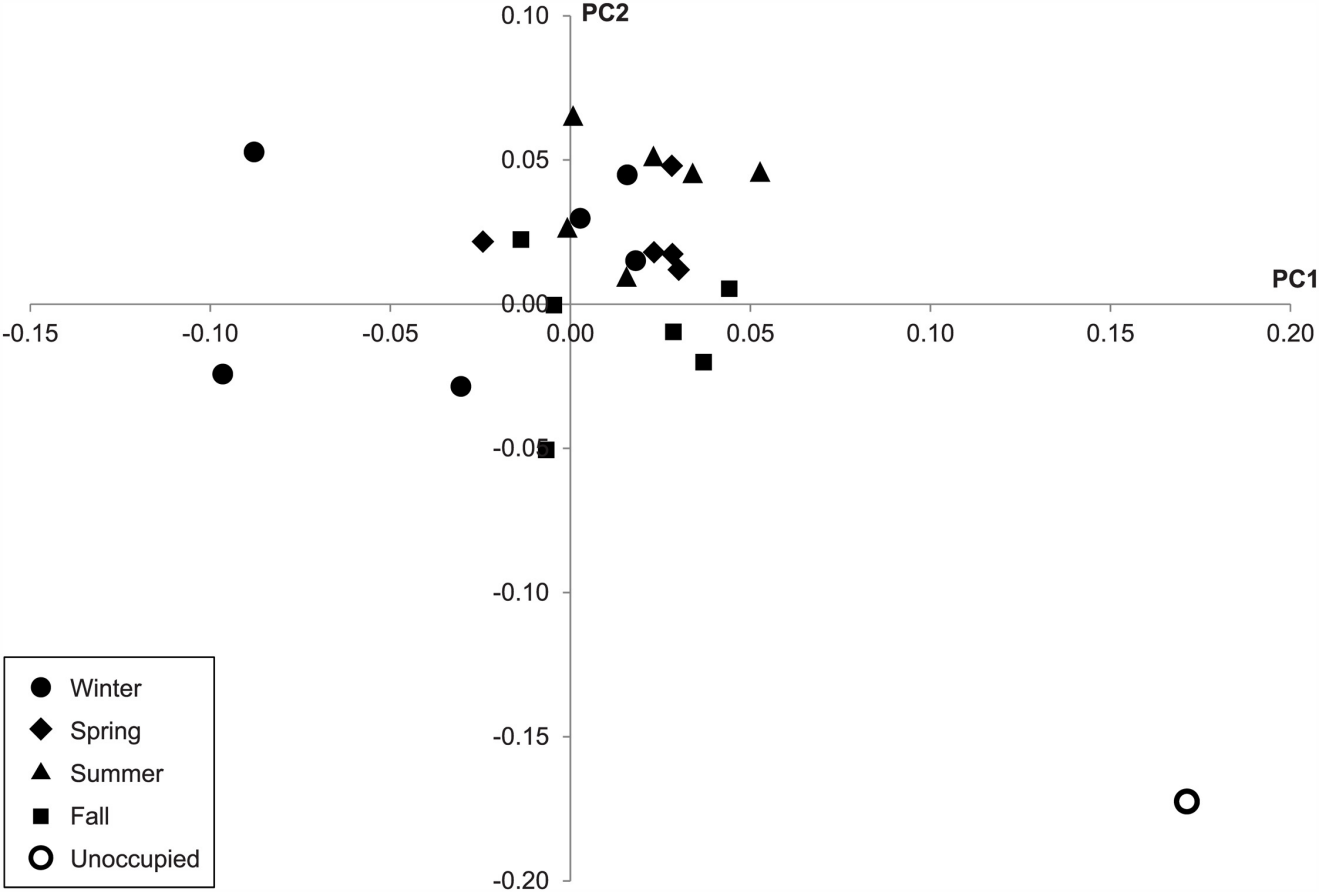
PCoA analysis of weighted UniFrac distance showing individual samples by season and one unoccupied sample.

Fig 2 shows principal component analysis of weighted UniFrac distance for each sample, grouped by season except for the sole sample collected when the daycare center was closed and unoccupied during the winter holidays (white dot in Fig 2). The unoccupied sample exhibited a different community structure compared to samples collected when the daycare center was open. The relative abundances of Gammaproteobacteria and Flavobacteriia were higher in the unoccupied sample compared to the occupied samples, while the relative abundances of Actinobacteria, Bacilli, and Betaproteobacteria were lower (Fig 1). Weighted UniFrac distance was significantly different between occupied and unoccupied conditions (p = 0.031), but unweighted UniFrac distance was not (p = 0.203). Kembel et al. [6] observed a slight increase in the relative abundance of Gammaproteobacteria and a decrease in the relative abundances of Betaproteobacteria and Bacilli in outdoor air compared to indoor air of a mechanically ventilated building. These results suggest that the bacterial community is more influenced by the outdoor environment when the daycare center is closed, due to the decrease in human occupancy.

According to principal component analysis, there were no apparent differences in the community structure of each sample between spring, summer, fall, and winter, as shown in Fig 2. Alpha diversity, as measured by number of OTUs, showed no seasonal trend. A more in-depth statistical analysis using ANOSIM (Table in S2 Table) suggested there to be a significant difference between seasons as measured using weighted UniFrac distance (p<0.05), with the exception of the winter-spring pair. On the other hand, analysis using unweighted UniFrac distance demonstrated no significant difference between seasons (p>0.05) with the exception of the winter-summer and spring-summer comparisons (p<0.05). These results suggest OTUs within the daycare microbial community to be largely consistent across seasons, but with varying relative abundances.

Using ADONIS, we evaluated the correlation between temperature, relative humidity, and air velocity in the HVAC duct and weighted UniFrac beta diversity. For the variables of temperature and relative humidity, the correlation analysis indicated a significant correlation (p = 0.02 and 0.005, respectively); however, the R^2^ values were 0.067 and 0.08, respectively, so the correlation explained only a small amount of variance. There was no significant correlation with air velocity in the duct (p = 0.673).

Previous work examining seasonal dynamics of microbes in indoor air has focused mainly on the concentrations of viable bacteria and fungi. Reponen et al. [58] showed a seasonal effect on the concentration of viable fungi in household air, while Moschandreas et al. [59] suggested that summer and fall produce the highest concentrations of cultivable bacteria in the air of homes. Due to the seasonality of both ambient conditions and certain diseases [41], we expected to observe seasonal differences in the community structure, but pathogens account for a small fraction of total bacteria. We also expected to observe seasonal differences because Bowers et al. [60] showed that the community structure in outdoor air is strongly affected by season. However, it appears that building occupants strongly influenced the microbiome of the daycare center’s air, obscuring any seasonality that might have existed in bacteria originating from outdoor air. In studying the seasonal dynamics of bacteria in dust collected from office buildings, Rintala et al. [53] also did not find clear differences in microbial community structure between seasons.

These results suggest that human occupancy, and the shedding of skin cells, is a major factor in determining the microbial community structure of airborne bacteria; and in heavily occupied environments such as daycare centers, schools, and offices, human occupancy has greater influence than does the outdoor environment in shaping the airborne microbiome. It is possible that in a very well-ventilated and low-occupancy environment, seasonal differences in the community structure of airborne microorganisms will be observed. This topic is an excellent avenue for future research.

### 3.2 Dynamics of Selected Viruses

Using PCR, we determined the presence or absence of adenovirus, enterovirus-71, IAV, RSV, rhinovirus, and rotavirus in samples based on whether an amplicon was produced at the same size as a positive control. Table 3 shows results for adenovirus, RSV, and rotavirus, where a plus sign indicates the virus was present based on a PCR positive result for the sampling period. We were not able to reach definite conclusions for enterovirus-71, IAV, or rhinovirus because PCR results indicated contamination on the HVAC filters for these viruses. Although two of our negative controls, the PCR water blank and the extraction kit blank, did not produce any PCR products, the unexposed filter did produce an amplicon at the correct size for the region being amplified. This suggests the possibility that the HVAC filters were contaminated with enterovirus-71, IAV, and rhinovirus and that the contamination was present on the unexposed filter and did not originate from the PCR reaction or extraction kit. Upon further investigation, we learned that the HVAC filters were not machine-produced but were mounted in the cardboard frame by hand, thus providing a possible avenue for contamination with human-associated microbes. It is critical that any future studies using PCR on bioaerosols collected from HVAC and other types of filters attempt to remove pre-existing nucleic acid by subjecting them to ultraviolet radiation, bleach, or some other decontamination method immediately prior to sampling.

**Table 3:** Presence (+) or absence (-) of a virus during each sampling period based on production of amplicon during PCR.

Sampling Start Date	Sampling End Date	RSV	Rotavirus	Adenovirus
20 January 2014	03 February 2014	-	-	-
03 February 2014	17 February 2014	-	-	-
17 February 2014	03 March 2014	-	-	-
03 March 2014	17 March 2014	-	-	-
17 March 2014	31 March 2014	+	-	-
31 March 2014	14 April 2014	+	-	-
14 April 2014	28 April 2014	+	-	-
28 April 2014	12 May 2014	-	-	-
12 May 2014	27 May 2014	+	-	-
27 May 2014	09 June 2014	+	-	-
09 June 2014	23 June 2014	+	-	-
23 June 2014	07 July 2014	+	-	-
07 July 2014	21 July 2014	+	-	-
21 July 2014	04 August 2014	-	-	-
04 August 2014	18 August 2014	-	-	-
18 August 2014	02 September 2014	-	-	-
02 September 2014	15 September 2014	-	-	-
15 September 2014	29 September 2014	-	-	-
29 September 2014	13 October 2014	-	-	-
13 October 2014	27 October 2014	-	-	-
27 October 2014	10 November 2014	+	-	-
10 November 2014	24 November 2014	+	-	-
24 November 2014	08 December 2014	+	-	-
08 December 2014	23 December 2014	+	-	-
23 December 2014	05 January 2015	+	-	-
05 January 2015	19 January 2015	+	-	-
19 January 2015	02 February 2015	+	-	-
Negative Control: PCR Water Blank		-	-	-
Negative Control: Extraction Kit Blank		-	-	-
Negative Control: Unexposed Filter		-	-	-
Positive Control		+	+	+

We detected RSV during two extended periods: 17 March 2014–21 July 2014 and 27 October 2014–2 February 2015 (Table 3). RSV is a lower respiratory tract viral pathogen in infants and young children that causes common-cold-like symptoms [61]. Lindsley et al. [62] showed that RSV occurred in aerosols small enough to remain airborne for an extended time, be transmitted via the airborne route, and cause infection. RSV is a seasonal virus that typically first appears in the northern hemisphere in November with an epidemic period lasting approximately four months [63]. In the southern hemisphere RSV typically first begins to cause infection in March or April. Our study did not collect any human information (e.g., health, travel, etc.), so we cannot confirm whether there was an out-of-season outbreak at the daycare center in the spring and early summer. Seasonality does appear to play a role in shaping the presence of RSV in the air of a daycare center.

We did not detect adenovirus and rotavirus at any time throughout the year (Table 3). This finding was contrary to expectations for adenovirus, as Fairchok et al. [32] suggested the virus causes 22% of respiratory tract infections in daycare centers. Airborne transmission of adenovirus in the environment has been demonstrated [64, 65], and the virus has been found in the air of a pediatric department of a hospital [66]. Further studies are needed to explain why the virus was not detected. Rotavirus causes a significant amount of diarrheal diseases in daycare centers [67, 68], and Dennehy et al. [69] detected rotavirus in 75% of air samples collected in hospital rooms with rotavirus-infected patients, even though the virus is shed in feces and is primarily transmitted via the fecal-oral route through contaminated hands, surfaces, and fomites [70, 71]. The daycare center we sampled has very strict policies regarding sanitization after diaper changes, washing hands frequently, and immediately sending home children with diarrhea, so the opportunities for aerosolization may have been minimal. Babies and toddlers do not use toilets, which can aerosolize fecal pathogens [72–74]; soiled diapers go directly into the trash. Additionally, the rotavirus vaccine became routine in 2006 in the United States [75, 76]. The vaccine is highly effective, reducing hospitalizations and emergency department visits by more than 80%.

We did not collect any human-subject related information (e.g., health records, travel information, etc.); however, in the future it would be valuable to correlate disease outbreaks in a daycare center with the airborne microbial community. Additionally, future studies should investigate microbial communities in the air, on surfaces, and in/on humans simultaneously at a daycare center; the continuing decrease in sequencing costs would make such a study feasible. Initially, the goal of our study was to examine viral communities using metagenomics; however, we were unable to obtain useful sequence data, most likely due to sampling in a limited-biomass environment (i.e., the air compared to water or soil). Using metagenomics to examine airborne viral communities has remained elusive due to challenges in both sampling and data analysis [43, 44].

## 4. Conclusions

We examined the airborne bacterial community and specific viruses collected on HVAC filters in a daycare center over a full year. Bacteria associated with human skin dominated the samples. The community structure differed when the center was occupied v. unoccupied, supporting the idea that human occupancy plays a critical role in shaping the indoor air microbiome. Seasonal dynamics were not evident in the bacterial community structure. Airborne RSV was present during wintertime, matching the typical seasonal pattern of the disease; RSV was also present in the spring through early summer. We did not detect adenovirus or rotavirus at any time. As we found HVAC filters to be contaminated with certain virus species, we urge future researchers to attempt to sterilize the filters prior to sampling.

## Supporting Information

S1 Table PCR cycling conditions used to detect the presence of specific viral bioaerosols throughout the year. (PDF)

S2 Table Pairwise comparisons of UniFrac distance by season (p-values resulting from 999 permutations). (PDF)

## References

[1] Klepeis NE, Nelson WC, Ott WR, Robinson JP, Tsang AM, Switzer P, et al The National Human Activity Pattern Survey (NHAPS): a resource for assessing exposure to environmental pollutants. J Expo Anal Env Epid. 2001;11(3): 231–52. 10.1038/sj.jea.7500165 11477521

[2] Cho I, Blaser MJ. The human microbiome: at the interface of health and disease. Nat Rev Genet. 2012;13(4): 260–70. 10.1038/nrg3182 22411464 PMC3418802

[3] Pflughoeft KJ, Versalovic J. Human microbiome in health and disease. Annu Rev Pathol. 2012;7: 99–122. Epub 2011/09/14. 10.1146/annurev-pathol-011811-132421 . 21910623

[4] Fujimura KE, Demoor T, Rauch M, Faruqi AA, Jang S, Johnson CC, et al House dust exposure mediates gut microbiome Lactobacillus enrichment and airway immune defense against allergens and virus infection. P Natl Acad Sci USA. 2014;111(2): 805–10. 10.1073/pnas.1310750111 PMC3896155 24344318

[5] Hospodsky D, Qian J, Nazaroff WW, Yamamoto N, Bibby K, Rismani-Yazdi H, et al Human occupancy as a source of indoor airborne bacteria. PLoS One. 2012;7(4): e34867 10.1371/journal.pone.0034867 22529946 PMC3329548

[6] Kembel SW, Jones E, Kline J, Northcutt D, Stenson J, Womack AM, et al Architectural design influences the diversity and structure of the built environment microbiome. ISME J. 2012;6(8): 1469–79. 10.1038/ismej.2011.211 22278670 PMC3400407

[7] Kembel SW, Meadow JF, O’Connor TK, Mhuireach G, Northcutt D, Kline J, et al Architectural design drives the biogeography of indoor bacterial communities. PLoS ONE. 2014;9(1): e87093 10.1371/journal.pone.0087093 24489843 PMC3906134

[8] Leung MHY, Wilkins D, Li EKT, Kong FKF, Lee PKH. Indoor-air microbiome in an urban subway network: diversity and dynamics. Appl Environ Microb. 2014;80(21): 6760–70. 10.1128/aem.02244-14 PMC4249038 25172855

[9] Meadow J, Altrichter A, Kembel S, Kline J, Mhuireach G, Moriyama M, et al Indoor airborne bacterial communities are influenced by ventilation, occupancy, and outdoor air source. Indoor Air. 2014;24(1): 41–8. 10.1111/ina.12047 23621155 PMC4285785

[10] Qian J, Hospodsky D, Yamamoto N, Nazaroff WW, Peccia J. Size-resolved emission rates of airborne bacteria and fungi in an occupied classroom. Indoor Air. 2012;22(4): 339–51. 10.1111/j.1600-0668.2012.00769.x 22257156 PMC3437488

[11] Wilkins D, Leung MHY, Lee PKH. Indoor air bacterial communities in Hong Kong households assemble independently of occupant skin microbiomes. Environ Microbiol. 2015; 10.1111/1462-2920.12889 25923292

[12] United States Census Bureau. Child Care an Important Part of American Life. 2013.

[13] Bell DM, Gleiber DW, Mercer AA, Phifer R, Guinter RH, Cohen AJ, et al Illness associated with child day care: a study of incidence and cost. Am J Public Health. 1989;79(4): 479–84. PMC1349981. 2929808 10.2105/ajph.79.4.479 PMC1349981

[14] Brady MT. Infectious disease in pediatric out-of-home child care. Am J Infect Control. 2005;33(5): 276–85. 15947744 10.1016/j.ajic.2004.11.007

[15] Haskins R, Kotch J. Day care and illness: evidence, cost, and public policy. Pediatrics. 1986;77(6 Pt 2): 951–82. Epub 1986/06/01. . 3012455

[16] Peerbooms PG, Engelen MN, Stokman DA, van Benthem BH, van Weert M-L, Bruisten SM, et al Nasopharyngeal carriage of potential bacterial pathogens related to day care attendance, with special reference to the molecular epidemiology of Haemophilus influenzae. J Clin Microbiol. 2002;40(8): 2832–6. 12149338 10.1128/JCM.40.8.2832-2836.2002 PMC120656

[17] Thacker SB, Addiss DG, Goodman RA, Holloway BR, Spencer HC. Infectious diseases and injuries in child day care: opportunities for healthier children. JAMA. 1992;268(13): 1720–6. 1527882

[18] Holmes SJ, Morrow AL, Pickering LK. Child-care practices: effects of social change on the epidemiology of infectious diseases and antibiotic resistance. Epidemiol Rev. 1996;18(1): 10–28. Epub 1996/01/01. . 8877328 10.1093/oxfordjournals.epirev.a017913

[19] Hurwitz ES, Gunn WJ, Pinsky PF, Schonberger LB. Risk of respiratory illness associated with day-care attendance: a nationwide study. Pediatrics. 1991;87(1): 62–9. 1984620

[20] Marbury MC, Maldonado G, Waller L. Lower respiratory illness, recurrent wheezing, and day care attendance. Am J Resp Crit Care. 1997;155(1): 156–61. 10.1164/ajrccm.155.1.9001305 9001305

[21] Pickering LK, Bartlett AV, Woodward WE. Acute infectious diarrhea among children in day care: epidemiology and control. Rev Infect Dis. 1986;8(4): 539–47. 3529310 10.1093/clinids/8.4.539

[22] Cauchemez S, Bhattarai A, Marchbanks TL, Fagan RP, Ostroff S, Ferguson NM, et al Role of social networks in shaping disease transmission during a community outbreak of 2009 H1N1 pandemic influenza. P Natl Acad Sci USA. 2011;108(7): 2825–30. 10.1073/pnas.1008895108 PMC3041067 21282645

[23] Ferguson NM, Cummings DAT, Fraser C, Cajka JC, Cooley PC, Burke DS. Strategies for mitigating an influenza pandemic. Nature. 2006;442(7101): 448–52. 16642006 10.1038/nature04795 PMC7095311

[24] Gojovic MZ, Sander B, Fisman D, Krahn MD, Bauch CT. Modelling mitigation strategies for pandemic (H1N1) 2009. Can Med Assoc J. 2009;181(10): 673–80. 10.1503/cmaj.091641 PMC2774362. 19825923 PMC2774362

[25] Huang SS, Finkelstein JA, Lipsitch M. Modeling community- and individual-level effects of child-care center attendance on pneumococcal carriage. Clin Infect Dis. 2005;40(9): 1215–22. 10.1086/428580 15825020

[26] Milne GJ, Kelso JK, Kelly HA, Huband ST, McVernon J. A small community model for the transmission of infectious diseases: comparison of school closure as an intervention in individual-based models of an influenza pandemic. PLoS ONE. 2008;3(12): e4005 10.1371/journal.pone.0004005 19104659 PMC2602849

[27] Cauchemez S, Valleron A-J, Boelle P-Y, Flahault A, Ferguson NM. Estimating the impact of school closure on influenza transmission from sentinel data. Nature. 2008;452(7188): 750–4. 10.1038/nature06732 18401408

[28] Couch RB, Cate TR, Douglas RG Jr, Gerone PJ, Knight V. Effect of route of inoculation on experimental respiratory viral disease in volunteers and evidence for airborne transmission. Bacteriol Rev. 1966;30(3): 517 5920335 10.1128/br.30.3.517-529.1966 PMC378233

[29] Roy CJ, Milton DK. Airborne transmission of communicable infection-the elusive pathway. DTIC Document, 2004. 10.1056/NEJMp048051 15102996

[30] Herfst S, Schrauwen EJ, Linster M, Chutinimitkul S, de Wit E, Munster VJ, et al Airborne transmission of influenza A/H5N1 virus between ferrets. Science. 2012;336(6088): 1534–41. 10.1126/science.1213362 22723413 PMC4810786

[31] Couch R, Douglas R, Lindgren K, Gerone P, Knight V. Airborne transmission of respiratory infection with coxsackievirus A type 21. Am J Epidemiol. 1970;91(1): 78–86. 5415578 10.1093/oxfordjournals.aje.a121115

[32] Fairchok MP, Martin ET, Chambers S, Kuypers J, Behrens M, Braun LE, et al Epidemiology of viral respiratory tract infections in a prospective cohort of infants and toddlers attending daycare. J Clin Virol. 2010;49(1): 16–20. 10.1016/j.jcv.2010.06.013 20650679 PMC7108368

[33] Ford-Jones EL, Wang E, Petric M, Corey P, Moineddin R, Fearon M. Rotavirus-associated diarrhea in outpatient settings and child care centers. Arch Pediat Adol Med. 2000;154(6): 586–93. 10.1001/archpedi.154.6.586 10850505

[34] Ferson M, Bell S. Outbreak of Coxsackievirus A16 hand, foot, and mouth disease in a child day-care center. Am J Public Health. 1991;81(12): 1675 1746672 10.2105/ajph.81.12.1675 PMC1405294

[35] Loda FA, Glezen WP, Clyde WA. Respiratory disease in group day care. Pediatrics. 1972;49(3): 428–37. 4334560

[36] Yang W, Elankumaran S, Marr LC. Concentrations and size distributions of airborne influenza A viruses measured indoors at a health centre, a day-care centre and on aeroplanes. J R Soc Interface. 2011;8(61): 1176–84. 10.1098/rsif.2010.0686 21300628 PMC3119883

[37] Prussin AJ, Garcia EB, Marr LC. Total concentrations of virus and bacteria in indoor and outdoor air. Environ Sci Technol Lett. 2015;2(4): 84–8. 26225354 10.1021/acs.estlett.5b00050 PMC4515362

[38] Shin S-K, Kim J, Ha S-m, Oh H-S, Chun J, Sohn J, et al Metagenomic insights into the bioaerosols in the indoor and outdoor environments of childcare facilities. PLoS ONE. 2015;10(5): e0126960 10.1371/journal.pone.0126960 26020512 PMC4447338

[39] Lee L, Tin S, Kelley ST. Culture-independent analysis of bacterial diversity in a child-care facility. BMC Microbiol. 2007;7(1): 1–13. 10.1186/1471-2180-7-27 17411442 PMC1853100

[40] Ansari SA, Springthorpe VS, Sattar SA. Survival and vehicular spread of human rotaviruses: possible relation to seasonality of outbreaks. Rev Infect Dis. 1991;13(3): 448–61. 1866549 10.1093/clinids/13.3.448

[41] Grassly NC, Fraser C. Seasonal infectious disease epidemiology. P Roy Soc B-Biol Sci. 2006;273(1600): 2541–50. 10.1098/rspb.2006.3604 PMC1634916 16959647

[42] Hii YL, Rocklov J, Ng N. Short term effects of weather on hand, foot and mouth disease. PLoS One. 2011;6(2): e16796 10.1371/journal.pone.0016796 21347303 PMC3037951

[43] Prussin AJ II, Marr LC, Bibby KJ. Challenges of studying viral aerosol metagenomics and communities in comparison with bacterial and fungal aerosols. FEMS Microbiol Lett. 2014;357(1): 1–9. 10.1111/1574-6968.12487 24891293

[44] Behzad H, Gojobori T, Mineta K. Challenges and opportunities of airborne metagenomics. Genome Biol Evol. 2015;7(5): 1216–26. 10.1093/gbe/evv064 25953766 PMC4453059

[45] Melling A, Whitelaw JH. Turbulent flow in a rectangular duct. J Fluid Mech. 1976;78(2): 289–315.

[46] Farnsworth JE, Goyal SM, Kim SW, Kuehn TH, Raynor PC, Ramakrishnan M, et al Development of a method for bacteria and virus recovery from heating, ventilation, and air conditioning (HVAC) filters. J Environ Monitor. 2006;8(10): 1006–13. 10.1039/b606132j 17240906

[47] Caporaso JG, Lauber CL, Walters WA, Berg-Lyons D, Huntley J, Fierer N, et al Ultra-high-throughput microbial community analysis on the Illumina HiSeq and MiSeq platforms. ISME J. 2012;6(8): 1621–4. 10.1038/ismej.2012.8 22402401 PMC3400413

[48] Caporaso JG, Kuczynski J, Stombaugh J, Bittinger K, Bushman FD, Costello EK, et al QIIME allows analysis of high-throughput community sequencing data. Nat Methods. 2010;7(5): 335–6. 10.1038/nmeth.f.303 20383131 PMC3156573

[49] DeSantis TZ, Hugenholtz P, Larsen N, Rojas M, Brodie EL, Keller K, et al Greengenes, a chimera-checked 16S rRNA gene database and workbench compatible with ARB. Appl Environ Microb. 2006;72(7): 5069–72. 10.1128/AEM.03006-05 PMC1489311 16820507

[50] Andersson A, Weiss N, Rainey F, Salkinoja-Salonen M. Dust-borne bacteria in animal sheds, schools and children's day care centres. J Appl Microbiol. 1999;86(4): 622–34. 10212408 10.1046/j.1365-2672.1999.00706.x

[51] Barberan A, Dunn RR, Reich BJ, Pacifici K, Laber EB, Menninger HL, et al The ecology of microscopic life in household dust. Proc R Soc B. 2015;282: 20151139 10.1098/rspb.2015.1139 PMC4571696 26311665

[52] Dannemiller KC, Gent JF, Leaderer BP, Peccia J. Influence of housing characteristics on bacterial and fungal communities in homes of asthmatic children. Indoor Air. 2015;in press: 10.1111/ina.12205 PMC4591094 25833176

[53] Rintala H, Pitkäranta M, Toivola M, Paulin L, Nevalainen A. Diversity and seasonal dynamics of bacterial community in indoor environment. BMC Microbiol. 2008;8(1): 56. 18397514 10.1186/1471-2180-8-56 PMC2323381

[54] Adams RI, Bateman AC, Bik HM, Meadow JF. Microbiota of the indoor environment: a meta-analysis. Microbiome. 2015;3(1): 1–18. 10.1186/s40168-015-0108-3 26459172 PMC4604073

[55] Grice EA, Segre JA. The skin microbiome. Nat Rev Microbiol. 2011;9(4): 244–53. 10.1038/nrmicro2537 21407241 PMC3535073

[56] Milstone LM. Epidermal desquamation. J Dermatol Sci. 2004;36(3): 131–40. 15541634 10.1016/j.jdermsci.2004.05.004

[57] Adams RI, Bhangar S, Pasut W, Arens EA, Taylor JW, Lindow SE, et al Chamber bioaerosol study: outdoor air and human occupants as sources of indoor airborne microbes. PloS One. 2015;10(5): e0128022 10.1371/journal.pone.0128022 26024222 PMC4449033

[58] Reponen T, Nevalainen A, Jantunen M, Pellikka M, Kalliokoski P. Normal range criteria for indoor air bacteria and fungal spores in a subarctic climate. Indoor Air. 1992;2(1): 26–31.

[59] Moschandreas D, Pagilla K, Storino L. Time and space uniformity of indoor bacteria concentrations in Chicago area residences. Aerosol Sci Technol. 2003;37(11): 899–906.

[60] Bowers RM, Sullivan AP, Costello EK, Collett JL, Knight R, Fierer N. Sources of bacteria in outdoor air across cities in the midwestern United States. Appl Environ Microb. 2011;77(18): 6350–6. 10.1128/AEM.05498-11 PMC3187178 21803902

[61] Mclntosh K. Respiratory syncytial virus Viral Infections of Humans: Springer; 1997 p. 691–711.

[62] Lindsley WG, Blachere FM, Davis KA, Pearce TA, Fisher MA, Khakoo R, et al Distribution of airborne influenza virus and respiratory syncytial virus in an urgent care medical clinic. Clin Infect Dis. 2010;50(5): 693–8. 10.1086/650457 20100093

[63] Stensballe LG, Devasundaram JK, Simoes EA. Respiratory syncytial virus epidemics: the ups and downs of a seasonal virus. Pediatr Infect Dis J. 2003;22(2): S21–S32. 12671449 10.1097/01.inf.0000053882.70365.c9

[64] Russell KL, Broderick MP, Franklin SE, Blyn LB, Freed NE, Moradi E, et al Transmission dynamics and prospective environmental sampling of adenovirus in a military recruit setting. J Infect Dis. 2006;194(7): 877–85. 16960774 10.1086/507426 PMC7109706

[65] Echavarria M, Kolavic SA, Cersovsky S, Mitchell F, Sanchez JL, Polyak C, et al Detection of adenoviruses (AdV) in culture-negative environmental samples by PCR during an AdV-associated respiratory disease outbreak. J Clin Microbiol. 2000;38(8): 2982–4. 10921963 10.1128/jcm.38.8.2982-2984.2000 PMC87165

[66] Wan G-H, Huang C-G, Huang Y-C, Huang J-P, Yang S-L, Lin T-Y, et al Surveillance of airborne adenovirus and Mycoplasma pneumoniae in a hospital pediatric department. PLoS One. 2012;7(3): e33974 10.1371/journal.pone.0033974 22470502 PMC3309934

[67] Waters V, Ford-Jones EL, Petric M, Fearon M, Corey P, Moineddein R, et al Etiology of community-acquired pediatric viral diarrhea: a prospective longitudinal study in hospitals, emergency departments, pediatric practices and child care centers during the winter rotavirus outbreak, 1997 to 1998. Pediatr Infect Dis J. 2000;19(9): 843–8. 11001107 10.1097/00006454-200009000-00007

[68] Ferson M, Stringfellow S, McPhie K, McIver C, Simos A. Longitudinal study of rotavirus infection in child-care centres. J Paediatr Child H. 1997;33(2): 157–60. 10.1111/j.1440-1754.1997.tb01020.x 9145361

[69] Dennehy PH, Nelson SM, Crowley BA, Saracen CL. Detection of rotavirus RNA in hospital air samples by polymerase chain reaction (PCR)• 828. Pediatr Res. 1998;43: 143.

[70] Dennehy PH. Transmission of rotavirus and other enteric pathogens in the home. Pediatr Infect Dis J. 2000;19(10): S103–S5. 11052397 10.1097/00006454-200010001-00003

[71] Sattar SA, Lloyd-Evans N, Springthorpe VS, Nair RC. Institutional outbreaks of rotavirus diarrhoea: potential role of fomites and environmental surfaces as vehicles for virus transmission. J Hyg. 1986;96(02): 277–89. 3701042 10.1017/s0022172400066055 PMC2129638

[72] Barker J, Jones MV. The potential spread of infection caused by aerosol contamination of surfaces after flushing a domestic toilet. J Appl Microbiol. 2005;99(2): 339–47. 10.1111/j.1365-2672.2005.02610.x 16033465

[73] Johnson DL, Mead KR, Lynch RA, Hirst DVL. Lifting the lid on toilet plume aerosol: A literature review with suggestions for future research. Am J Infect Control. 2013;41(3): 254–8. 10.1016/j.ajic.2012.04.330 23040490 PMC4692156

[74] Verani M, Bigazzi R, Carducci A. Viral contamination of aerosol and surfaces through toilet use in health care and other settings. Am J Infect Control. 2014;42(7): 758–62. 10.1016/j.ajic.2014.03.026 24818773 PMC7132667

[75] Glass RI, Parashar UD. Rotavirus vaccines—balancing intussusception risks and health benefits. New Engl J Med. 2014;370(6): 568–70. 10.1056/NEJMe1315836 24422677 PMC5716463

[76] Vesikari T, Matson DO, Dennehy P, Van Damme P, Santosham M, Rodriguez Z, et al Safety and efficacy of a pentavalent human—bovine (WC3) reassortant rotavirus vaccine. New Engl J Med. 2006;354(1): 23–33. 16394299 10.1056/NEJMoa052664

[77] Sarantis H, Johnson G, Brown M, Petric M, Tellier R. Comprehensive detection and serotyping of human adenoviruses by PCR and sequencing. J Clin Microbiol. 2004;42(9): 3963–9. 15364976 10.1128/JCM.42.9.3963-3969.2004 PMC516336

[78] Yang X, Li G, Wen K, Bui T, Liu F, Kocher J, et al A neonatal gnotobiotic pig model of human enterovirus 71 infection and associated immune responses. Emerg Microbes Infect. 2014;3(5): e35 10.1038/emi.2014.35 26038741 PMC4051366

[79] Borg I, Rohde G, Löseke S, Bittscheidt J, Schultze-Werninghaus G, Stephan V, et al Evaluation of a quantitative real-time PCR for the detection of respiratory syncytial virus in pulmonary diseases. Eur Respir J. 2003;21(6): 944–51. 12797486 10.1183/09031936.03.00088102

[80] Gambarino S, Costa C, Elia M, Sidoti F, Mantovani S, Gruosso V, et al Development of a RT real-time PCR for the detection and quantification of human rhinoviruses. Mol Biotechnol. 2009;42(3): 350–7. 10.1007/s12033-009-9164-x 19291427 PMC7091102

[81] McKell AO, Nichols JC, McDonald SM. PCR-based approach to distinguish group A human rotavirus genotype 1 vs. genotype 2 genes. J Virol Methods. 2013;194(1): 197–205. 24012969 10.1016/j.jviromet.2013.08.025

