# Supplementary material for: Seasonal Dynamics of the Airborne Bacterial Community and Selected Viruses in a Children’s Daycare Center
Source: PLoS One. 2016 Mar 4;11(3):e0151004. doi: 10.1371/journal.pone.0151004 (PMC4778917; doi:10.1371/journal.pone.0151004)
Supplement: S1 Table — (PDF) [file pone.0151004.s001.pdf]

Seasonal Dynamics of the Airborne Bacterial Community and Selected Viruses in a Children's Daycare Center

Aaron J. Prussin II, Amit Vikram, Kyle J. Bibby, and Linsey C. Marr

S1 Table. PCR cycling conditions used to detect the presence of specific viral bioaerosols throughout the year.

Virus	Initial Denature (Time)	Denature (Time)	Anneal (Time)	Extension (Time)	Repeat	Final Extension (Time)
Adenovirus	95 °C (3 min)	95 °C (60 s)	51 °C (60 s)	72 °C (60 s)	37 cycles	72 °C (10 min)
Enterovirus-71	95°C (3 min)	95 °C (30 s)	56 °C (30 s)	72 °C (60 s)	37 cycles	72 °C (10 min)
IAV	95 °C (3 min)	95 °C (30 s)	55 °C (30 s)	72 °C (60 s)	37 cycles	72 °C (10 min)
RSV	95 °C (3 min)	95 °C (30 s)	55 °C (30 s)	72 °C (60 s)	37 cycles	72 °C (10 min)
Rhinovirus	95 °C (3 min)	95 °C (30 s)	55 °C (30 s)	72 °C (60 s)	37 cycles	72 °C (10 min)
Rotavirus	95 °C (3 min)	95 °C (30 s)	48 °C (30 s)	72 °C (60 s)	37 cycles	72 °C (10 min)
