# Supplementary material for: Seasonal Dynamics of the Airborne Bacterial Community and Selected Viruses in a Children’s Daycare Center
Source: PLoS One. 2016 Mar 4;11(3):e0151004. doi: 10.1371/journal.pone.0151004 (PMC4778917; doi:10.1371/journal.pone.0151004)
Supplement: S2 Table — (PDF) [file pone.0151004.s002.pdf]

Seasonal Dynamics of the Airborne Bacterial Community and Selected Viruses in a Children's Daycare Center

Aaron J. Prussin II, Amit Vikram, Kyle J. Bibby, and Linsey C. Marr

S2 Table. Pairwise comparisons of UniFrac distance by season (p-values resulting from 999 permutations).

Weighted UniFrac

	Winter	Spring	Summer	Fall
**Winter**				
**Spring**	0.051			
**Summer**	0.003	0.001		
**Fall**	0.002	0.001	0.002	

Unweighted UniFrac

	Winter	Spring	Summer	Fall
**Winter**				
**Spring**	0.102			
**Summer**	0.004	0.019		
**Fall**	0.127	0.062	0.171	
